# Genetic Evidence Supporting the Repurposing of mTOR Inhibitors for Reducing BMI

**DOI:** 10.3390/biomedicines13040839

**Published:** 2025-03-31

**Authors:** Ping Peng, Fan Shen, Bi Peng, Ziqi Chen, Lei Zhou, Xingjie Hao, Yuanhui Liu

**Affiliations:** 1 Department of Oncology, Tongji Hospital, Tongji Medical College, Huazhong University of Science and Technology, Wuhan 430030, China; pengbi668@163.com (B.P.); chenziqi_dr@163.com (Z.C.); zhoulei2010@tjh.tjmu.edu.cn (L.Z.); 2 Nursing Department, Tongji Hospital, Tongji Medical College, Huazhong University of Science and Technology, Wuhan 430030, China; fanfan@tjh.tjmu.edu.cn; 3 Department of Epidemiology and Biostatistics, School of Public Health, Tongji Medical College, Huazhong University of Science and Technology, Wuhan 430030, China; xingjie@hust.edu.cn

**Keywords:** mTOR inhibitor, genetic evidence, repurposing opportunity, BMI

## Abstract

**Background:** Although mTOR has long been regarded as a promising target for cancer treatment, the efficacy of mTOR inhibitors in most clinical trials has been rather limited. Nevertheless, their favorable safety profile has opened up opportunities for drug repurposing, even as their potential applications across various diseases remain largely unexplored. **Methods:** We performed an MR-PheWAS analysis across 1431 phenotypes to explore drug repurposing opportunities. We analyzed GWAS data of 452 plasma metabolites, 731 immune traits, and 412 gut microbiota to uncover potential mechanisms for the causal link between the mTOR gene and body mass index (BMI). **Results:** A causal link between mTOR gene expression and BMI has been established. Additionally, mTOR-related vulnerabilities associated with BMI, including alterations in metabolites, immune traits, and gut microbiota, were identified. **Conclusions:** The identified causal relationship between mTOR and BMI suggests novel potential non-cancer applications for mTOR inhibitors.

## 1. Introduction

Rapamycin was first discovered in 1964 as a compound with antifungal, immunosuppressive, and antitumor properties [1]. In 1994, the mammalian target of rapamycin (mTOR) was identified as its direct target [2]. Since then, mTOR has been extensively studied, with many studies demonstrating that its activity is often abnormally elevated in cancer [3]. And several generations of mTOR inhibitors have been developed, showing great potential in cancer therapy. However, despite over 500 clinical trials testing mTOR inhibitors alone or in combination with other drugs, few have yielded significant clinical benefits [4,5,6,7,8].

While most of the mTOR inhibitors have not achieved their primary endpoints in cancer clinical trials, they offer promising drug repurposing opportunities in the treatment of non-cancer diseases, as they have already undergone safety and tolerability evaluations and are ready for clinical use. In fact, there have been many successful examples of drug repurposing, such as using anti-diabetic medications for cancer [9]. Similarly, mTOR inhibitors have been employed as immunosuppressants in organ transplantation and as anti-aging drugs. However, other potential applications of mTOR inhibitors remain largely unknown.

Mendelian randomization (MR) is an approach that investigates the associations between naturally occurring genetic variants in drug targets and disease outcomes [10]. This method is less prone to the conventional issue of confounding, as germline genetic variants are randomly allocated during meiosis. Additionally, MR analysis allows for the assessment of the long-term effects of drug target modulation. By leveraging the availability of phenotype GWAS data, we performed a MR-phenome-wide association study (PheWAS) to explore possible non-cancer uses of mTOR inhibitors.

The PheWAS results established a causal link to between mTOR gene expression and body mass index (BMI), which is confirmed across different consortiums. Given that mTOR is a key regulator of cell metabolism and immune cell proliferation and function, we utilized GWAS data on 452 plasma metabolites and 731 immune traits to conduct MR analyses, identifying causal links between mTOR-related metabolites and immune traits. Additionally, we identified mTOR-related gut microbiota, as these interact with both metabolites and immune cells. We examined the mediation effect of metabolite, immune, and microbiota traits on the mTOR-BMI link to provide potential mechanistic insight.

## 2. Methods

### 2.1. Study Design

**Figure 1:**
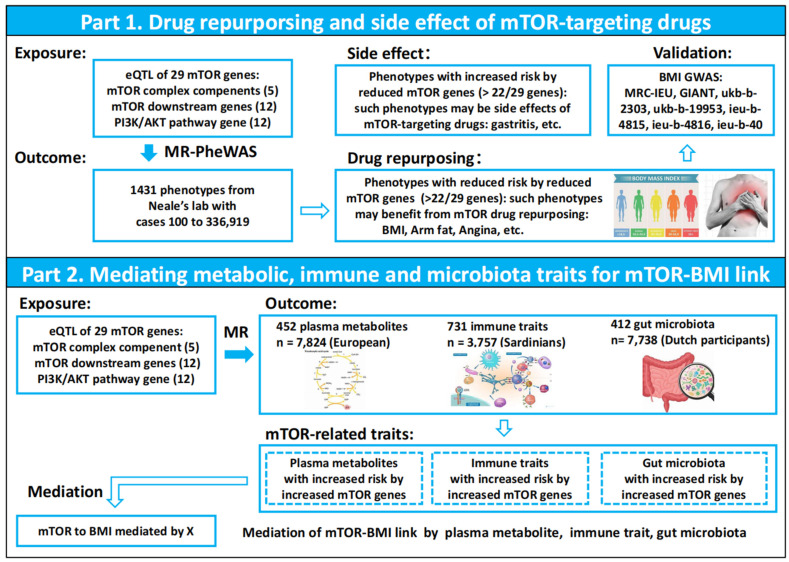
Flowchart of this study.

Figure 1 describes the design of study and the workflow of the selection of genetic variants and analytical methods. The mTOR complex component genes, as well as the mTOR downstream and feedback-activated PI3K/AKT pathway genes, were extracted from a literature review. To generate eQTL instruments for the mentioned genes, genetic variants located within 1000 kb on either side of the coding sequence (in cis) that are robustly associated with gene expression were extracted using eQTLs summary statistics obtained from the eQTLGen Consortium [11]. The eQTLGen Consortium includes information on 10,317 trait-associated single-nucleotide polymorphisms (SNPs) derived from 31,684 individuals across 37 datasets. From cis-eQTL, 36,859 SNPs, 79,832 SNPs, and 84,930 SNPs associated with the expression of 5 mTOR complex component genes, 12 mTOR downstream genes, and 12 feedback activated PI3K/AKT pathway genes, respectively, were selected. A total of 6 BMI GWAS datasets were included, and the details are presented in Table S1. The relationships between 29 mTOR genes and 1431 phenotypes were systematically analyzed using the two-sample mendelian randomization (MR) method, identifying and validating a causal link between mTOR genes and BMI. Genome-wide association studies (GWASs) of 452 plasma metabolites, 731 immune traits, and 412 gut microbiota profiles were leveraged to explore the potential mechanisms underlying this causal link.

The SNPs were matched to the human genome Build 37 (NCBI GRCh37) to ensure consistent genomic coordinates. We used the “TwoSampleMR” package to conduct MR analysis. The analyses were performed using R software version 4.2.2.

### 2.2. Phenotype Data Source and MR-PheWAS Analysis

A total of 1431 disease-related risk factor GWAS datasets from Neale’s lab (https://www.nealelab.is/uk-biobank, accessed 1 August 2024) were selected for the MR-PheWAS analysis. A *p*-value of less than 0.05 and a Bonferroni-corrected *p*-value were used as thresholds to identify nominal and corrected significant causal relationships.

### 2.3. 452 Plasma Metabolites GWAS Data Source

The GWAS data for metabolites were sourced from a study by Shin et al. in 2023 [12]. The researchers conducted a GWAS on 452 metabolites in human blood, analyzing 7824 adults from two European population cohorts.

### 2.4. 731 Immune Cell Phenotype GWAS Data Source

GWAS data for immune phenotypes were obtained from the GWAS Catalog (accession numbers GCST0001391 to GCST0002121) [13]. The study analyzed 731 immunophenotypes in total, including 192 relative cell counts (RCs), 118 absolute cell counts (ACs), 389 median fluorescence intensities (MFIs), and 32 morphological parameters (MPs). Genotyping was performed using four Illumina arrays (Illumina, San Diego, CA, USA), covering approximately 22 million SNPs, with imputation based on a Sardinian sequence-based reference panel.

### 2.5. 412 Gut Microbiota GWAS Data Source

SNPs associated with the composition of the human gut microbiome were selected as instrumental variables (IVs) from a series of GWAS datasets [14]. This analysis, based on 7738 participants from the Dutch Microbiome Project, integrated 16S ribosomal RNA gene sequencing profiles with genotyping data to investigate the relationship between human genetic variation and the gut microbiome. In total, 207 taxa and 205 pathways representing microbial composition were examined.

### 2.6. Mediation Analysis

A two-step MR analysis employing mediation analysis was conducted to explore whether plasma metabolites, immune cell phenotypes, and gut microbiota mediate the causal pathway from mTOR gene to BMI. The total effect was decomposed into a mediating effect and a direct effect. The mediating effect of traits on BMI was further broken down into (i) the causal effect of the exposure on the mediator (beta1) and (ii) the causal effect of the mediator on the outcome (beta2). The formula used for calculating the mediating effect is as follows: mediating effect = beta1 × beta2. The mediated proportion was calculated using the following formula: mediated proportion = mediating effect/total effect.

### 2.7. Role of the Funding Source

The funder of this study had no role in the study design, data collection, analysis, interpretation, or writing of this manuscript.

### 2.8. Ethics

All of the studies included in this research had been approved by the corresponding ethical review committees, and all participants signed the consent forms.

## 3. Results

### 3.1. MR-PheWAS Analysis Identified a Causal Relationship Between mTOR and BMI, Highlighting Potential Opportunities for Drug Repurposing

**Figure 2:**
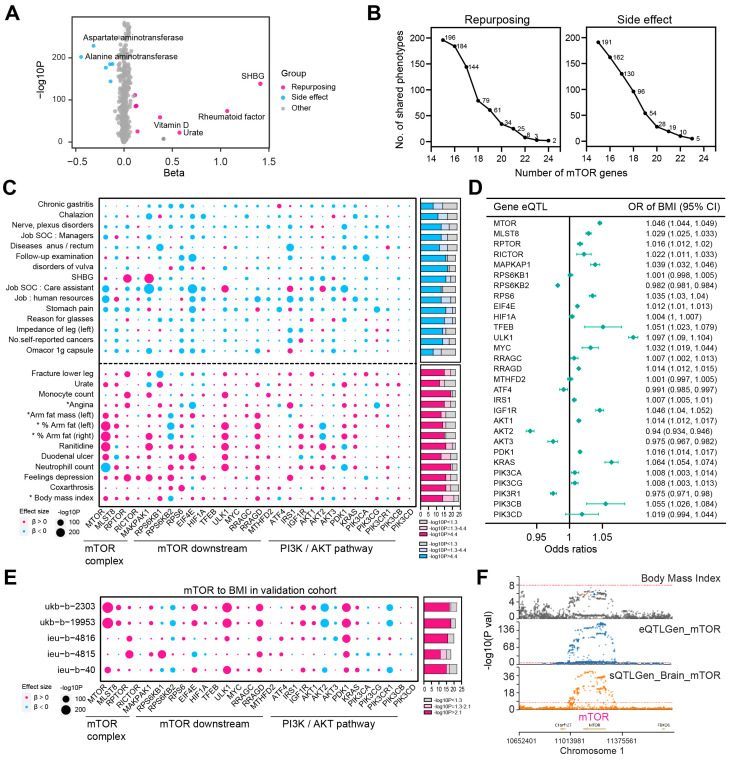
MR-PheWAS analysis identified the drug repurposing opportunities and side effects of mTOR targeting drugs. (**A**) Volcano plot showing the effect of mTOR gene expression on the risk of 1431 phenotypes. The data are presented as beta scores (*X* axis) and −log10P values (*Y* axis) from the MR-PheWAS analysis. Red dots represent phenotypes where mTOR expression was significantly positively associated with increased risk (β > 0.1, *p* < 0.05/1431), indicating potential targets for repurposing through mTOR inhibition. Blue dots represent phenotypes where increased mTOR expression was significantly inversely associated with risk (β < 0, *p* < 0.05/1431), suggesting potential side effects when inhibiting mTOR. (**B**) Number of phenotypes with consistent direction of effect size (β > 0, repurposing, left panel; β < 0, side effect, right panel) causally linked to mTOR genes. We identified 2 phenotypes that were positively associated with the expression of 24 out of 29 mTOR genes. Additionally, 3 and 8 phenotypes were linked to the expression of 23 and 22 mTOR genes, respectively (left panel). Conversely, the risk of 5 and 10 phenotypes was inversely associated with the increased expression of 23 and 22 mTOR genes, respectively (right panel). (**C**) Bubble plot showing the effect size direction and significance (−log10P) of the MR-PheWAS results for all 29 mTOR genes. Red dots represent positive effect sizes (β > 0), while blue dots represent negative effect sizes (β < 0). The phenotypes that were causally (or inversely causally) related to more than 22 of the 29 mTOR genes are shown. Notably, the expression of 24 out of 29 mTOR genes was associated with increased BMI. The accompanying bar plot illustrates the number of mTOR genes significantly associated with each phenotype, using Bonferroni-corrected significance (*p* < 0.05/1431, −log10P > 4.4, red in bottom panel, blue in top panel) or nominal significance (*p* < 0.05, −log10P > 1.3, light red in bottom panel, light blue in top panel). The phenotypes marked with an asterisk are associated with metabolism. (**D**) Forest plot showing the MR results linking the expression of 29 mTOR genes to BMI. eQTL, expression quantitative trait loci. (**E**) Bubble plot showing the causal link between the expression of 29 mTOR genes and BMI, validated using five additional GWAS datasets. The accompanying bar plot illustrates the number of mTOR genes significantly associated with each phenotype, with Bonferroni-corrected significance (−log10P > 4.4, red) and nominal significance (−log10P > 1.3, light red). (**F**) Locus zoom plots illustrating consistent genetic effects from body mass index (BMI) GWAS and cis-eQTL and sQTL near mTOR. The top plot displays the −log10 (*p* value) from the BMI GWAS. The second plot shows the −log10 (*p* value) for SNP associations with mTOR gene expression (cis-eQTL). The third plot shows the −log10 (*p* value) for SNP associations with mTOR splicing in the brain (sQTL).

To identify potential drug repurposing opportunities for mTOR inhibitors, we conducted a MR-Phenome-wide association (MR-PheWAS) analysis to systematically assess the causal relationship between gene expression of 29 mTOR genes (5 mTOR complex components and 24 mTOR downstream and PI3K/AKT pathway genes) and 1431 phenotypes from Neale’s lab dataset. The analysis revealed that the genetically elevated mTOR gene level was associated with increased sex hormone-binding globulin (SHBG), a disorder implicated in obesity and insulin resistance (Figure 2A) [15]. To strengthen our findings, we ranked the phenotypes based on the number of mTOR genes involved, hypothesizing that phenotypes with consistent causal links to multiple mTOR genes would present more robust opportunities for drug repurposing. Similarly, phenotypes showing reduced risk (β < 0) associated with the expression of multiple mTOR genes were considered potential side effects of mTOR inhibitor use (Figure 2B,C, Table S2). Notably, body mass index (BMI) was causally linked to the expression of 24 out of 29 mTOR genes, with 21 of these links being statistically significant (Figure 2B–D). In addition to BMI, other metabolic-related phenotypes, such as % arm fat (right), % arm fat (left), arm fat mass (left), and angina, were ranked among the most promising repurposing opportunities (Figure 2C). To further validate the robustness of the mTOR-BMI link, we conducted MR analysis using additional BMI GWAS from the MRC-IEU and GIANT consortium. The causal relationship between mTOR and BMI was consistently confirmed across these datasets (Figure 2E). We performed colocalization analysis to explore the overlap of SNPs within the mTOR gene and those most strongly associated with BMI. Notably, we identified a leading SNP on chromosome 1 shared between mTOR gene expression and BMI, as well as another SNP shared between mTOR gene splicing in the brain and BMI (Figure 2F).

### 3.2. mTOR-Related Metabolites and Their Mediating Role in the mTOR-BMI Link

**Figure 3:**
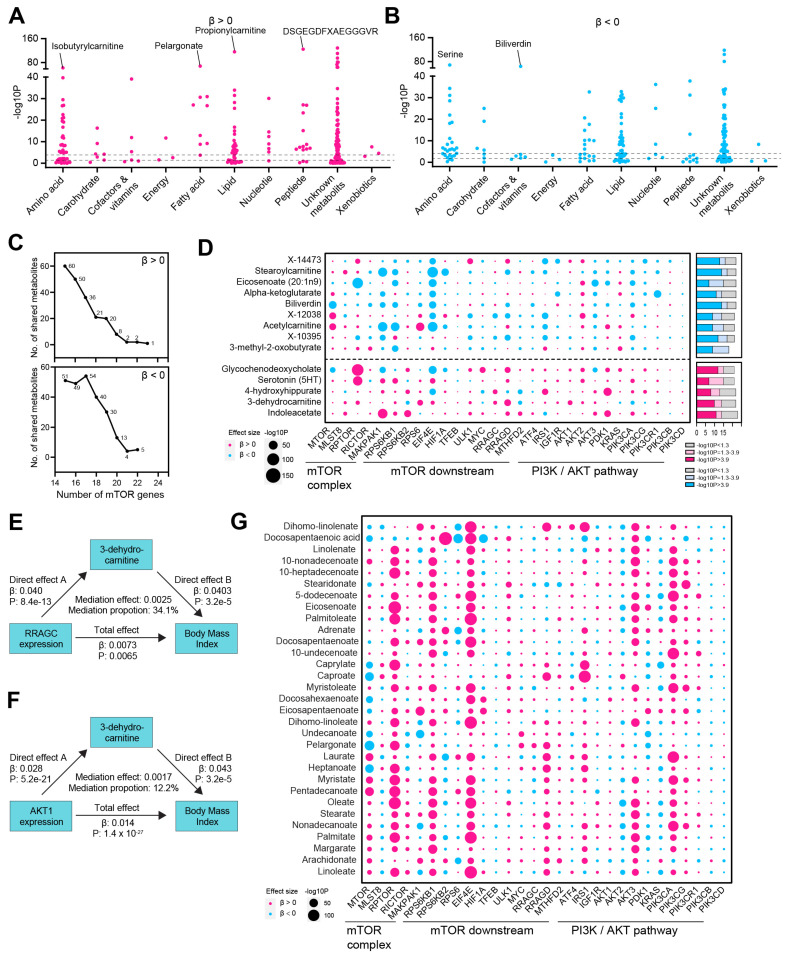
Mediation effect of metabolites in causal link between mTOR and BMI. (**A**,**B**) Manhattan dot plot displays the MR results assessing the causal relationship between mTOR expression and plasma metabolites. The significance (−log10P) values for metabolites across various groups are shown, with metabolites either positively (β > 0, (**A**)) or inversely (β < 0, (**B**)) linked to mTOR expression. (**C**) Number of metabolites with consistent direction of effect size (β > 0, upper panel; β < 0, bottom panel) causally linked to mTOR genes. We identified 1 metabolite that was positively associated with the expression of 23 out of 29 mTOR genes. Additionally, 2 and 2 phenotypes were linked to the expression of 22 and 21 mTOR genes, respectively (upper panel). Conversely, the risk of 5 and 4 metabolites was inversely associated with the increased expression of 22 and 21 mTOR genes, respectively (bottom panel). (**D**) Bubble plot showing the effect size direction and significance (−log10P) of the MR results for all 29 mTOR genes and 452 plasma metabolites. Red dots represent positive effect sizes (β > 0), while blue dots represent negative effect sizes (β < 0). The metabolites that were causally (or inversely causally) related to more than 21 of the 29 mTOR genes are shown. The accompanying bar plot illustrates the number of mTOR genes significantly associated with each metabolite, using Bonferroni-corrected significance (*p* < 0.05/452, −log10P > 3.9; red in the bottom panel; blue in the top panel) or nominal significance (*p* < 0.05, −log10P > 1.3; light red in the bottom panel; light blue in the top panel). (**E**) The mediating role of 3-dehydro-carnitine in the relationship between RRAGC expression and BMI. (**F**) The mediating role of 3-dehydro-carnitine in the relationship between AKT1 expression and BMI. (**G**) The bubble plot depicts the direction of effect size and significance (−log10P) of the MR analysis results for 29 mTOR genes and 31 fatty acids. Red dots indicate positive effect sizes (β > 0), while blue dots represent negative effect sizes (β < 0).

Our findings established a causal link between mTOR and BMI. Given mTOR’s regulatory role in metabolism [16], we aimed to investigate whether mTOR-influenced metabolites mediate the relationship between mTOR and BMI. First, we aimed to identify plasma metabolites that are either positively or inversely associated with genetically increased expression of mTOR genes, which we refer to as mTOR-related metabolites. The genetic elevation of mTOR expression exhibited a positive causal effect on several metabolites, including isobutyrylcarnitine (an amino acid), pelargonate (a fatty acid), propionylcarnitine (a lipid), and DSGEGDFXAEGGGVR (a peptide), among others (Figure 3A). Conversely, it showed an inverse causal effect on serine (an amino acid) and biliverdin (a cofactor and vitamin) (Figure 3B). Expanding the analysis to all 29 included mTOR genes, we identified one metabolite that was positively associated with the expression of 23 mTOR genes (Figure 3C, Table S3). Additionally, two metabolites were linked to the expression of 22 and 21 mTOR genes, respectively. In contrast, the expression of 22 and 21 mTOR genes was inversely associated with five and four metabolites, respectively (Figure 3C,D). Given that BMI is primarily associated with lipid metabolism, particularly fatty acid metabolism, we evaluated the relationship between mTOR genes and metabolites within the fatty acid metabolism pathway. As anticipated, mTOR genes exhibited a positive causal relationship with 31 fatty acid metabolites (Figure 3G). To explore the potential mechanism by which mTOR regulates BMI through metabolites, we performed a mediation analysis. Specifically, the causal effects of mTOR genes, including RRAGC and AKT1 on BMI, were mediated by 3-dehydrocarnitine, with mediation effects of 34.1% (*p* = 0.005) and 12.2% (*p* = 0.011), respectively (Figure 3E,F).

### 3.3. The mTOR-BMI Association Was Partially Mediated by Immune Traits

**Figure 4:**
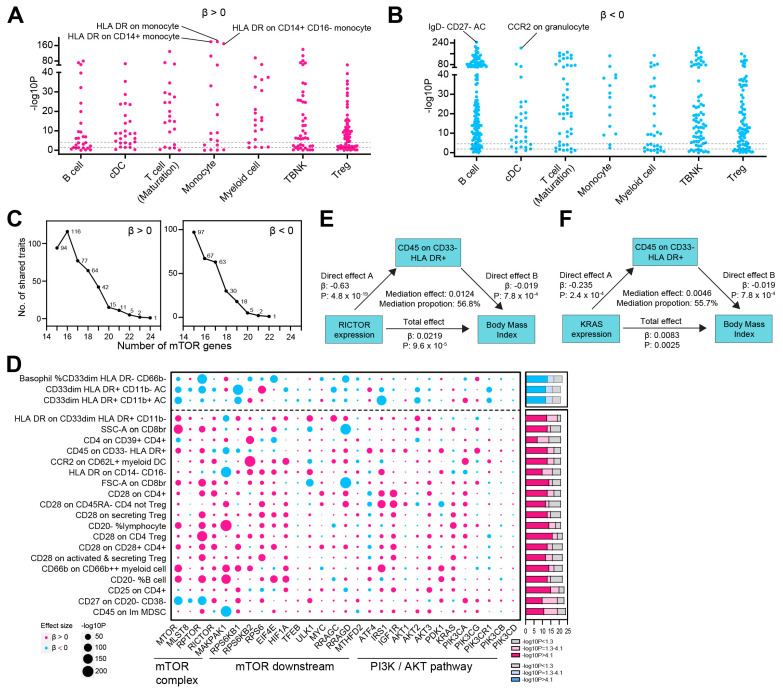
Mediation effect of immune trait in causal link between mTOR and BMI. (**A**,**B**) Manhattan dot plot displays the MR results assessing the causal relationship between mTOR expression and immune phenotypes. The significance (−log10P) values for immune phenotypes across various groups are shown, with trait either positively (β > 0, (**A**)) or inversely (β < 0, (**B**)) linked to mTOR expression. (**C**) Number of immune traits with consistent direction of effect size (β > 0, upper panel; β < 0, bottom panel) causally linked to mTOR genes. We identified 1 immune trait that was positively associated with the expression of 24 out of 29 mTOR genes. Additionally, 2, 5, and 11 immune traits were linked to the expression of 23, 22, and 21 mTOR genes, respectively (upper panel). Conversely, the risk of 1 and 2 immune traits was inversely associated with the increased expression of 22 and 21 mTOR genes, respectively (bottom panel). (**D**) The left bubble plot showing the effect size direction and significance (−log10P) of the MR results for causal links between 29 mTOR genes and 731 immune traits. Red dots represent positive effect sizes (β > 0), while blue dots represent negative effect sizes (β < 0). The immune traits that were causally (or inversely causally) related to more than 21 of the 29 mTOR genes are shown. The accompanying bar plot illustrates the number of mTOR genes significantly associated with each immune trait, using Bonferroni-corrected significance (*p* < 0.05/731, −log10P > 4.1; red in the bottom panel; blue in the top panel) or nominal significance (*p* < 0.05, −log10P > 1.3; light red in the bottom panel; light blue in the top panel). (**E**) The mediating role of CD45 on CD33- HLA DR+ in the relationship between RICTOR expression and BMI. (**F**) The mediating role of CD45 on CD33-HLA DR+ in the relationship between KRAS expression and BMI.

Given that mTOR plays a major role in immune cell growth and activation [17], we further examined the causal relationship between mTOR genes and 731 immune traits [13]. We first observed that the genetic proxies of mTOR expression were most significantly correlated with ‘HLA DR on monocyte’, ‘HLA DR on CD14+ monocyte’, and ‘HLA DR on CD14+ CD16- monocyte’ (Figure 4A). On the contrary, mTOR expression was inversely related with immune traits including ‘IgD-CD27-AC’, ‘CCR2 on granulocyte’ (Figure 4B). We further expanded our analysis to include all 29 mTOR genes. We identified one immune trait positively associated with the expression of 24 out of the 29 mTOR genes. Additionally, 2, 5, and 11 immune traits were linked to the expression of 23, 22, and 21 mTOR genes, respectively. Conversely, only 1 and 2 immune traits were inversely associated with the increased expression of 22 and 21 mTOR genes, respectively (Figure 4C,D, Table S4). Therefore, for 19 out of the 22 immune traits most strongly associated with mTOR—where the effect direction was consistent in more than 21 of the 29 mTOR genes—increased mTOR gene expression was linked to a higher risk of elevated immune trait levels (β > 0) (Figure 4D). We further checked the mediation effects of immune traits on the mTOR-BMI link. The causal effects of mTOR genes, including RICTOR and KRAS, on BMI were mediated with mediation effects of 56.8% (*p* < 0.05) and 55.7% (*p* < 0.05), respectively (Figure 4E,F).

### 3.4. Identification of mTOR-Related Gut Microbiota and Its Role in Mediating the mTOR-BMI Connection

**Figure 5:**
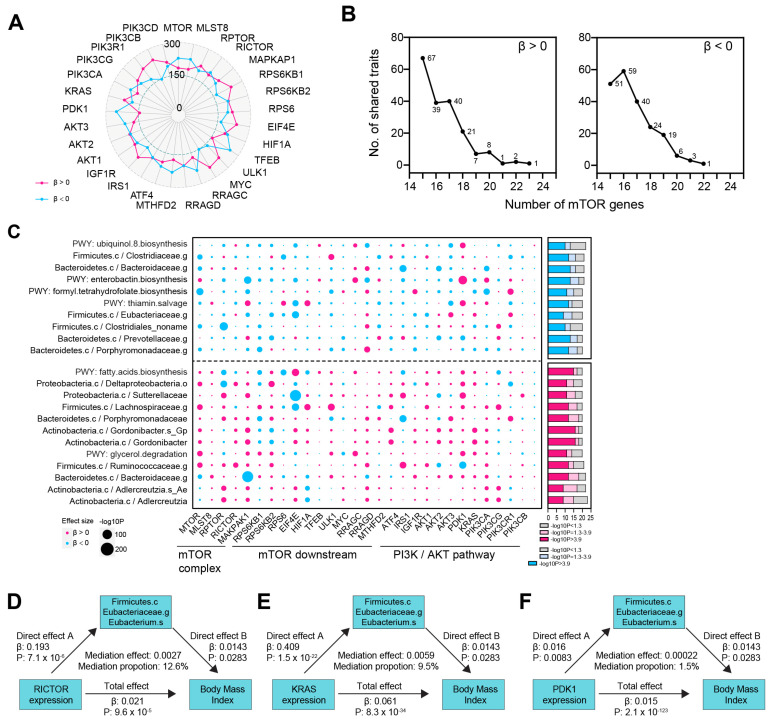
Mediation effect of gut microbiota in causal link between mTOR and BMI. (**A**) Radar plot showing the number of gut microbiota positively (red line) or inversely (blue line) influenced by each mTOR gene. (**B**) Number of gut microbiota with consistent direction of effect size (β > 0, upper panel; β < 0, bottom panel) causally linked to mTOR genes. We identified 1 gut microbiota that was positively associated with the expression of 23 out of 29 mTOR genes. Additionally, 2, 1, and 8 phenotypes were linked to the expression of 22, 21, and 20 mTOR genes, respectively (left panel). Conversely, the risk of 1, 3, and 6 gut microbiota were inversely associated with the increased expression of 22, 21, and 20 mTOR genes respectively (right panel). (**C**) The left bubble plot showing the effect size direction and significance (−log10P) of the MR results for causal links between 29 mTOR genes and 412 gut microbiota. Red dots represent positive effect sizes (β > 0), while blue dots represent negative effect sizes (β < 0). The gut microbiota that were causally (or inversely causally) related to more than 20 of the 29 mTOR genes are shown. The accompanying bar plot illustrates the number of mTOR genes significantly associated with each gut microbiota, using Bonferroni-corrected significance (*p* < 0.05/412, −log10P > 3.9; red in the bottom panel; blue in the top panel) or nominal significance (*p* < 0.05, −log10P > 1.3; light red in the bottom panel; light blue in the top panel). (**D**) The mediating role of Eubacterium.s in the relationship between RICTOR expression and BMI. (**E**) The mediating role of Eubacterium.s in the relationship between KRAS expression and BMI. (**F**) The mediating role of Eubacterium.s in the relationship between PDK1 expression and BMI.

The gut microbiota exerts its effects by secreting metabolites and influencing immune cells [18]. Given that mTOR has been previously shown to have a causal relationship with both metabolites and immune traits, we sought to investigate the link between mTOR genes and the gut microbiota. We assessed the causal association between 29 mTOR-related genes and 412 gut microbiota taxa [14] (Figure 5A, Table S5). Our analysis identified one gut microbiota species that was positively associated with the expression of 23 out of the 29 mTOR genes. Additionally, two, one, and eight microbial phenotypes were linked to the expression of 22, 21, and 20 mTOR genes, respectively. Conversely, the expression of 22, 21, and 20 mTOR genes was inversely associated with the risk of one, three, and six gut microbiota taxa, respectively (Figure 5B). Among these, the species k__Bacteria.p__Actinobacteria.c__Actinobacteria.o__Coriobacteriales.f__Coriobacteriaceae.g__Adlercreutzia.s ranked as the most positively associated with mTOR gene expression (Figure 5C). We further explored the role of gut microbiota in mediating the causal relationship between mTOR and BMI. Specifically, we found that k__Bacteria.p__Firmicutes.c__Clostridia.o__Clostridiales.f__Eubacteriaceae.g__Eubacterium.s__Eubacterium_hallii mediated the causal effects of the mTOR genes RICTOR, KRAS, and PDK1 on BMI (Figure 5D–F).

## 4. Discussion

Researchers and physicians have been investigating mTOR inhibitors as potential cancer treatments for over three decades, based on preclinical experiments and observational data. However, most randomized clinical trials have fallen short of expectations. In contrast, our Mendelian randomization (MR) study provides genetic evidence supporting the use of mTOR inhibitors in the treatment of non-cancer diseases. Our study is the first to establish a causal link between mTOR and BMI using GWAS data, providing large-scale population-based genetic evidence. More importantly, we demonstrate that this link is mediated by plasma metabolites, immune traits, and gut microbiota species, although these findings require further experimental validation.

Several factors contribute to the limitations of mTOR inhibitors in cancer treatment, including tumor heterogeneity, adaptive resistance mechanisms, and feedback activation of alternative signaling pathways [19]. Additionally, the complexity of mTOR signaling across different cancer types can impact drug efficacy. More importantly, mTOR inhibition slows tumor proliferation, leading to suboptimal responses when combined with chemotherapy, as chemotherapy primarily targets rapidly dividing cells [20]. In contrast, the mechanism by which mTOR inhibitors influence BMI reduction differs from their role in cancer. This distinction underscores the promise of mTOR inhibitors in non-cancer applications and highlights the importance of further mechanistic studies and validation.

Here, we explored the potential for repurposing mTOR inhibitors in non-cancer therapeutic applications as they have already undergone safety and tolerability evaluations and are ready for clinical use. Through PheWAS analysis, we identified a causal relationship between mTOR gene expression and BMI, which was further validated using five additional GWAS datasets for BMI. This finding suggests the potential use of mTOR inhibitors for non-cancer applications, in addition to their well-known roles in preventing immune rejection in organ transplantation and in anti-aging therapies.

In fact, mTOR inhibitors have shown promise in the treatment of several non-cancer diseases due to their role in regulating cellular processes such as metabolism, immune response, and cell growth. These diseases include autoimmune conditions [21], neurodegenerative diseases [22], and cardiovascular diseases [22]. Given mTOR’s involvement in regulating metabolism, mTOR inhibitors like rapamycin have also been explored for metabolic disorders such as type 2 diabetes and non-alcoholic fatty liver disease (NAFLD), where they may improve insulin sensitivity, lipid metabolism, and reduce inflammation.

Consistent with our findings, several retrospective and preclinical studies support the correlation between mTOR and BMI. In a cohort of kidney transplant patients, treatment with the mTOR inhibitor sirolimus resulted in a significantly lower BMI compared to patients receiving cyclosporine (24.17 ± 2.99 vs. 25.97 ± 5.01 kg/m^2^, *p* = 0.031) [23]. Another retrospective study found that individuals with hyperlipidemia exhibited elevated mTOR levels, which were positively correlated with insulin resistance, high blood pressure, and BMI [24]. One in vivo study demonstrated that rapamycin, an mTOR inhibitor, reduces food intake and fat mass in diet-induced obesity (DIO) mice [25]. Another study showed that rapamycin alleviates age-dependent obesity, which is associated with increased mTOR signaling in hypothalamic POMC neurons [26].

If mTOR is leveraged as a therapeutic target for BMI management in the future, its potential impact could be substantial. Given its pivotal role in nutrient sensing, metabolism, and energy regulation, targeting mTOR may offer a novel pharmacological approach to BMI modulation, complementing traditional lifestyle interventions such as diet and exercise. From a clinical standpoint, repurposing mTOR inhibitors for BMI reduction could provide a targeted strategy for obesity management, particularly in individuals with metabolic syndrome. However, due to the intricate and multifactorial nature of BMI regulation, further research is necessary to optimize dosing regimens, assess long-term safety, and evaluate potential adverse effects. Additionally, exploring personalized treatment strategies, such as genetic and metabolic profiling, may help identify individuals who are most likely to benefit from mTOR-targeted interventions, ultimately enhancing therapeutic precision and efficacy.

### Limitations of This Study

While we have demonstrated the potential of repurposing mTOR inhibitors for non-cancer applications and identified novel mTOR-related metabolites, immune traits, and gut microbiota, these findings need to be confirmed through experimental studies. Further research is required to validate the mechanisms underlying these associations and assess their clinical relevance.

Although this paper establishes a causal link between mTOR and BMI, it does not rule out the possibility of other pathways being linked to BMI. Investigating these potential connections would necessitate a transcriptome-wide association study (TWAS).

## 5. Conclusions

A causal relationship between mTOR genes and BMI has been established. Several mTOR-related metabolic, immune, and gut microbiota traits have been identified as potential novel cancer targets, though more extensive experimental studies and clinical trials are needed to confirm these findings.

## Data Availability

The data used in this study are publicly available for download and are listed in Table S1. The code required to reproduce the results in this article is available upon request.

## Supplementary Materials

The following supporting information can be downloaded at: www.mdpi.com/article/10.3390/biomedicines13040839/s1, Table S1. Included BMI GWAS studies. Table S2. MR results of causal links between the expression of 29 mTOR genes and 1431 phenotypes. Table S3. MR results of causal links between the expression of 29 mTOR genes and 452 plasma metabolites. Table S4. MR results of causal links between the expression of 29 mTOR genes and 731 immune traits. Table S5. MR results of causal links between the expression of 29 mTOR genes and 412 gut microbiota.
